# Supplementary material for: Longitudinal associations between stroke and psychosis: a population-based study
Source: Psychol Med. 2023 Jun 5;53(16):7698–706. doi: 10.1017/S0033291723001575 (PMC10755233; doi:10.1017/S0033291723001575)
Supplement: Richards-Belle et al. supplementary material [file S0033291723001575sup001.docx]

**Supplementary Material to**

**Longitudinal associations between stroke and psychosis: a population-based study**

Alvin Richards-Belle, Norman Poole, David P.J. Osborn, Vaughan Bell

**Table of contents**

[Supplementary Table 1. Comparison of participants with and without missing baseline alcohol use data. 2](#_Toc133916921)

[Supplementary Table 2. Stroke status and ever-reported psychiatric diagnoses for sample. 3](#_Toc133916922)

[Supplementary Table 3. Study participation across stroke and psychosis groups. 4](#_Toc133916923)

[Supplementary Table 4. Odds ratios estimates for stroke risk after psychosis and psychosis risk after stroke (complete-case). 5](#_Toc133916924)

[Supplementary Table 5. Hazard ratios estimates for stroke risk after psychosis and psychosis risk after stroke (complete-case). 6](#_Toc133916925)

[Supplementary Table 6. Schoenfeld global test *P* values. 7](#_Toc133916926)

| *Characteristic* | *With observed data,  N = 17,738* | *With missing data,  N = 2,070* |
| --- | --- | --- |
| Wave 1 Age | 58 (50, 68) | 48 (41, 55) |
| Sex, n (%) |  |  |
| *Male* | 7,941 (44.8%) | 1,072 (51.8%) |
| *Female* | 9,797 (55.2%) | 998 (48.2%) |
| Ethnicity, n (%) |  |  |
| *White* | 16,228 (91.5%) | 1,744 (84.3%) |
| *Non-White* | 593 (3.3%) | 251 (12.1%) |
| *Unknown* | 917 (5.2%) | 75 (3.6%) |
| Net financial wealth (quintile), n (%) | | |
| *5 (highest)* | 3,488 (20.1%) | 264 (13.2%) |
| *4* | 3,425 (19.7%) | 314 (15.7%) |
| *3* | 3,315 (19.1%) | 319 (15.9%) |
| *2* | 3,256 (18.7%) | 413 (20.6%) |
| *1 (lowest)* | 3,899 (22.4%) | 694 (34.6%) |
| Smoked cigarettes at baseline, n (%) | 3,157 (18.3%) | 386 (19.6%) |
| Level of vigorous activity at baseline, n (%) | | |
| *More than once a week* | 3,768 (21.2%) | 474 (25.8%) |
| *Once a week* | 1,772 (10.0%) | 172 (9.4%) |
| *One to three times a month* | 1,668 (9.4%) | 172 (9.4%) |
| *Hardly ever or never* | 10,527 (59.4%) | 1,020 (55.5%) |
| Region, n (%) |  |  |
| *East Midlands* | 1,735 (9.8%) | 236 (11.4%) |
| *East of England* | 2,136 (12.0%) | 209 (10.1%) |
| *London* | 1,661 (9.4%) | 227 (11.0%) |
| *North East* | 1,171 (6.6%) | 111 (5.4%) |
| *North West* | 2,276 (12.8%) | 312 (15.1%) |
| *South East* | 2,973 (16.8%) | 354 (17.1%) |
| *South West* | 2,009 (11.3%) | 177 (8.6%) |
| *Wales* | 1 (0.0%) | 0 (0.0%) |
| *West Midlands* | 1,878 (10.6%) | 252 (12.2%) |
| *Yorkshire and the Humber* | 1,892 (10.7%) | 189 (9.1%) |
| Reported stroke by 10y, n (%) | 1,160 (6.5%) | 119 (5.7%) |
| Reported psychosis by 10y, n (%) | 130 (0.7%) | 20 (1.0%) |

# Supplementary Table 1. Comparison of participants with and without missing baseline alcohol use data.

The level of missing data in baseline variables was low, with most either complete or with less than 5% missing values – the exception was level of alcohol use (10%). This level of missingness did not differ based on stroke/psychosis status, but participants with alcohol data tended to be younger, male, of non-white ethnicity and in the lowest quintile of net financial wealth. There were little differences according to region, smoking status, or physical activity categories.

|  | *No Stroke or Psychosis* | *Psychosis only* | *Stroke and Psychosis* | *Stroke only* |
| --- | --- | --- | --- | --- |
| N | 18,403 | 126 | 24 | 1,255 |
| Stroke |  |  |  |  |
| Age at first stroke, median (IQR) | - | - | 66 (58, 79) | 69 (59, 77) |
| Unknown | *-* | *-* | 2 | 114 |
| Stroke recurrences, mean (SD) | - | - | 2.2 (3.0) | 1.1 (1.5) |
| Stroke recurrences (if >0), mean (SD) | - | - | 3.1 (3.2) | 2.0 (1.5) |
| Unknown | *-* | *-* | 7 | 543 |
| Ever-reported psychiatric diagnoses |  |  |  |  |
| Depression, n (%) | 1,733 (9.4%) | 111 (88.1%) | 20 (83.3%) | 145 (11.6%) |
| Anxiety, n (%) | 1,496 (8.1%) | 104 (82.5%) | 18 (75.0%) | 129 (10.3%) |

# Supplementary Table 2. Stroke status and ever-reported psychiatric diagnoses for sample.

|  | *No Stroke or Psychosis* | *Psychosis only* | *Stroke and Psychosis* | *Stroke only* |
| --- | --- | --- | --- | --- |
| N | 18,403 | 126 | 24 | 1,255 |
| Number of waves participated^1^ | 4.0 (2.0, 7.0) | 5.0 (3.0, 7.0) | 6.0 (3.0, 8.0) | 4.0 (2.0, 6.0) |
| Participated in all waves, n (%) | 3,060 (16.6%) | 20 (15.9%) | 2 (8.3%) | 143 (11.4%) |
| Died before Wave 6, n (%) | 2,293 (12.5%) | 12 (9.5%) | 5 (20.8%) | 409 (32.6%) |

*^1^* Median (IQR)

# Supplementary Table 3. Study participation across stroke and psychosis groups.

|  | *Unadjusted* | *Adjusted, model 1* | *Adjusted, model 2* |
| --- | --- | --- | --- |
|  | OR (95% CI) | OR (95% CI) | OR (95% CI) |
| Odds of stroke in psychosis |  |  |  |
| At 4 years | 3.99 (2.39, 6.34) | 5.46 (3.18, 8.93) | 4.14 (2.20, 7.26) |
| At 10 years | 2.79 (1.76, 4.25) | 3.44 (2.12, 5.37) | 2.74 (1.57, 4.53) |
| Odds of psychosis in stroke |  |  |  |
| At 4 years | 3.99 (2.39, 6.34) | 5.00 (2.90, 8.24) | 3.62 (1.91, 6.43) |
| At 10 years | 2.79 (1.76, 4.25) | 3.26 (2.00, 5.11) | 2.53 (1.45, 4.19) |

*^1^* OR = Odds Ratio. All models were significant at the p<0.001 level. Model 1: adjusted for age, sex, ethnicity, quintile of net financial wealth + participant (frailty). Model 2: adjusted for age, sex, ethnicity, quintile of net financial wealth, smoking status, level of alcohol use in the past 12 months, level of vigorous physical activity + participant (frailty).

# Supplementary Table 4. Odds ratios estimates for stroke risk after psychosis and psychosis risk after stroke (complete-case).

|  | *Unadjusted* | *Adjusted, model 1* | *Adjusted, model 2* |
| --- | --- | --- | --- |
|  | HR (95% CI) | HR (95% CI) | HR (95% CI) |
| Stroke risk after psychosis | 2.87 (1.91, 4.29) | 3.82 (2.43, 6.02) | 3.40 (1.93, 5.98) |
| Psychosis risk after stroke | 3.08 (1.99, 4.77) | 4.79 (2.62, 8.77) | 5.35 (2.37, 12.1) |

*^1^* HR = Hazard Ratio. All models were significant at the p<0.001 level. Model 1: adjusted for age, sex, ethnicity, quintile of net financial wealth + participant (frailty). Model 2: adjusted for age, sex, ethnicity, quintile of net financial wealth, smoking status, level of alcohol use in the past 12 months, level of vigorous physical activity + participant (frailty).

# Supplementary Table 5. Hazard ratios estimates for stroke risk after psychosis and psychosis risk after stroke (complete-case).

|  | *P* |
| --- | --- |
| Psychosis in stroke |  |
| Unadjusted model | 0.48 |
| Model 1 | >0.99 |
| Model 2 | >0.99 |
| Stroke in psychosis |  |
| Unadjusted model | 0.40 |
| Model 1 | >0.99 |
| Model 2 | >0.99 |

# Supplementary Table 6. Schoenfeld global test *P* values.
